# Genomic Characterization of Clinical Extensively Drug-Resistant *Acinetobacter pittii* Isolates

**DOI:** 10.3390/microorganisms9020242

**Published:** 2021-01-25

**Authors:** Peechanika Chopjitt, Nuntiput Putthanachote, Ratchadaporn Ungcharoen, Rujirat Hatrongjit, Parichart Boueroy, Yukihiro Akeda, Kazunori Tomono, Shigeyuki Hamada, Anusak Kerdsin

**Affiliations:** 1 Faculty of Public Health, Chalermphrakiat Sakon Nakhon Campus, Kasetsart University, Sakon Nakhon 47000, Thailand; Ratchadaporn.un@ku.th (R.U.); parichart.bou@ku.th (P.B.); anusak.ke@ku.th (A.K.); 2 Clinical Microbiology Laboratory, Roi-Et Hospital, Roi-Et 45000, Thailand; nuntiput101@gmail.com; 3 Department of General Science, Faculty of Science and Engineering, Chalermphrakiat Sakon Nakhon Province Campus, Kasetsart University, Sakon Nakhon 47000, Thailand; rujirat.ha@ku.th; 4 Department of Infection Control and Prevention, Osaka University Graduate School of Medicine, 2-2 Yamadaoka, Suita, Osaka 565-0871, Japan; akeda@biken.osaka-u.ac.jp (Y.A.); tomono@hp-infect.med.osaka-u.ac.jp (K.T.); 5 Research Institute for Microbial Diseases, Osaka University, Osaka 565-0871, Japan; hamadas@biken.osaka-u.ac.jp

**Keywords:** *Acinetobacter pittii*, carbapenem-resistance, whole genome sequencing, extensively drug-resistant, XDR, Thailand

## Abstract

Carbapenem-resistant *Acinetobacter pittii* (CRAP) is a causative agent of nosocomial infections. This study aimed to characterize clinical isolates of CRAP from a tertiary hospital in Northeast Thailand. Six isolates were confirmed as extensively drug-resistant *Acinetobacter pittii* (XDRAP). The *bla*_NDM-1_ gene was detected in three isolates, whereas *bla*_IMP-14_ and *bla*_IMP-1_ were detected in the others. Multilocus sequence typing with the Pasteur scheme revealed ST220 in two isolates, ST744 in two isolates, and ST63 and ST396 for the remaining two isolates, respectively. Genomic characterization revealed that six XDRAP genes contained antimicrobial resistance genes: ST63 (A436) and ST396 (A1) contained 10 antimicrobial resistance genes, ST220 (A984 and A864) and ST744 (A56 and A273) contained 9 and 8 antimicrobial resistance genes, respectively. The single nucleotide polymorphism (SNP) phylogenetic tree revealed that the isolates A984 and A864 were closely related to *A. pittii* YB-45 (ST220) from China, while A436 was related to *A. pittii* WCHAP100020, also from China. A273 and A56 isolates (ST744) were clustered together; these isolates were closely related to strains 2014S07-126, AP43, and WCHAP005069, which were isolated from Taiwan and China. Strict implementation of infection control based upon the framework of epidemiological analyses is essential to prevent outbreaks and contain the spread of the pathogen. Continued surveillance and close monitoring with molecular epidemiological tools are needed.

## 1. Introduction

*Acinetobacter calcoaceticus-baumannii* complex (ACB complex) includes *A. baumannii*, *A. calcoaceticus*, *A. pittii*, *A. nosocomialis*, *A. seifertii*, and *A. dijkshoorniae* [1,2,3]. They are the primary bacteria causing nosocomial infection [1,2,3]. Among these, *A. baumannii* is known as the most clinically relevant and common nosocomial infection worldwide. So far, most studies have focused on *A. baumannii*, with relatively fewer studies on *A. pittii* because of its low prevalence and low rates of resistance in past decades. However, recently, *A. pitii* has shown increased carbapenem resistance and changes in its resistance mechanisms. Carbapenem-resistant *A. pittii* (CRAP) has been extensively reported and disseminated worldwide [4,5]. It is associated with human infection and intestinal carriage and is recognized as a significant cause of nosocomial infection in various countries, particularly in intensive care unit settings [1,4,5]. In Taiwan, the percentage of *A. pittii* increased by 4.6%, and the rates of resistance to carbapenems increased from 4.5% in 2010 to 9.3% and 25.8% in 2012 and 2014, respectively [6]. A study in a French hospital from January 2010 to December 2017 revealed 73 out of 120 cases were classified as hospital-acquired bacteraemia; 54.8% (*n* = 40) were associated with *A. pittii*, 39.7% (*n* = 29) were associated with *A. baumannii*, and 5.5% (*n* = 4) were associated *A. nosocomialis* [5].

Horizontal gene transfer is an important contributor to the spread of carbapenem-hydrolyzing class D β-lactamases (CHDLs) among other *Acinetobacter* species, and particularly of *A. pittii*, mainly in Asia [7]. Previously, OXA-58-like and metallo-β-lactamase (MBLs) were primarily responsible for CRAP, but *bla*_OXA-23-like_ and *bla*_OXA-24-like_ have recently become more common [6]. The major mechanisms of resistance in CRAP found in Thailand include production of OXA-23 and OXA-58 [7,8]. Apart from *bla*_OXA_ genes with MBLs, genes such as *bla*_IMP-14a_ have been reported in CRAP isolates from Thailand, while *bla*_NDM_-carrying organisms have been reported in countries like Malaysia, Taiwan, South Korea, Japan, and Brazil, but not in Thailand [4,6,8,9,10,11]. Genomic characterization of metallo-β-lactamase harboring *A. pittii* has not yet been investigated in the isolates from Thailand.

In this study, we characterized the antimicrobial susceptibility, resistance genes, plasmid typing, and genetic relationships of CRAP harboring *bla*_NDM_ and *bla*_IMP_ isolated from patients in Northeast Thailand. We demonstrated that almost all the CRAP isolates used in this study showed extensive drug resistance (XDR). In addition, all genomic sequences of extensively drug-resistant *Acinetobacter pittii* (XDRAP) strains were comparative analyzed.

## 2. Materials and Methods

### 2.1. Ethics

This study was reviewed and approved by the Roi Et Hospital Ethics Review board (ERB). The ethic approval number is 034/2560. The medical records of seven patients were reviewed by the attending physicians at the hospital using the clinical case record form approved by ERB. The ERB waived the requirement for informed consent for patient signatures; however, the attending physicians provided written informed consent for all cases as the study satisfied the conditions of the policy statement on ethical conduct for research involving humans. This study was conducted according to the principles of the Declaration of Helsinki.

### 2.2. Bacterial Identification

From April 2017 to March 2018, we established laboratory-based surveillance to determine carbapenem-resistant Gram-negative bacteria in an 800-bed tertiary-care hospital in Roi Et province, northeastern Thailand. A criterion in this study was that all carbapenem-resistant *Acinetobacter calcoaceticus-baumannii* complex (CRACB) were collected from any specimens during the surveillance program. A total of 832 nonrepetitive carbapenem-resistant ACB (CRACB) isolates were collected. Presumptive identification was performed at the hospital using a conventional biochemical test [12]. All CRACB isolates were sent to our laboratory to identify species levels using gyrB-multiplex PCR [13], and to confirm *A. baumannii* using PCR for the blaOXA-51-like gene, which is intrinsic of *A. baumannii* [14].

### 2.3. Antimicrobial Susceptibility Testing

Minimum inhibitory concentrations (MICs) of 13 antimicrobial agents—ceftazidime, cefepime, ceftriaxone, cefotaxime, doripenem, imipenem, meropenem, colistin, gentamicin, amikacin, netimicin, ciprofloxacin, and trimethoprim-sulfamethoxazole—were examined in the isolates using the Sensititre (Thermo Fisher Scientific, Cleveland, OH, USA). Disk diffusion with tetracycline, piperacillin, and piperacillin/tazobactam was also performed. Interpretation was performed according to the Clinical and Laboratory Standards Institute (CLSI, 2020) guidelines [15].

### 2.4. Detection of Antimicrobial Resistance Genes

Multiplex PCR was performed to detect oxacillinase (OXA), carbapenemase, and mobile colistin resistance genes including *bla*_OXA-23-like_, *bla*_OXA-24-like_, *bla*_OXA-51-like_, *bla*_OXA-58-like_, *bla*_OXA-10-like_, *bla*_IMP_, *bla*_NDM_, *bla*_OXA-48like_, *bla*_KPC_, and *mcr-*1 (Table S1) [16,17,18]. All PCR products were confirmed using Sanger sequencing by Apical Scientific (Sdn Bhd, Selangor, Malaysia).

### 2.5. PCR-Based Replicon Typing

The plasmid replicons were determined in all CRAP isolates by PCR-based replicon typing method (Table S2; [19]). The nineteen different homology groups (GRs) were detected based on similarities of nucleotide sequence in 27 replicase genes.

### 2.6. Multilocus Sequence Typing

Multilocus sequence typing (MLST) was performed according to the Pasteur scheme (https://pubmlst.org/abaumannii/) using seven housekeeping genes (*gltA*, *gryB*, *gdhB*, *recA*, *cpn60*, *rpoD*, and *gpi*). The sequence types (STs) were identified by comparing the allele sequences in the MLST database. A goeBURST analysis for sequence types was performed using the PHYLOViZ 2.0 program [20]. Construction of phylogenetic trees for all STs using concatenated sequences was performed using MEGA-X (version 10.1.7) software [21].

### 2.7. Whole-Genome Sequencing and Analysis

Genomic DNA of six XDR *A. pittii* isolates in this study was extracted using the E.Z.N.A.^®^ Tissue DNA Kit (OMEGA Bio-tek, Norcross, GA, USA) according to the manufacturer’s instructions. The quality of DNA was assessed using a Nanodrop 2000 Spectrophotometer (Thermo Fisher Scientific, Waltham, MA, USA) and by agarose gel electrophoresis. Whole-genome sequencing and assembled DNA sequence data were analyzed on the Illumina platform as described previously [22]. Confirmation of the species using the whole-genome sequences was done on KmerFinder 3.1 (https://cge.cbs.dtu.dk/services/KmerFinder/) [23,24]. Antimicrobial resistance genes were identified with ResFinder 3.1 (https://cge.cbs.dtu.dk/services/ResFinder/) [25], CARD version 2020 (https://card.mcmaster.ca/analyze/rgi) [26], and the BacWGSTdb 2.0 online tool [27,28]. Plasmid replicons were analyzed using PlasmidFinder 2.1 [29] (https://cge.cbs.dtu.dk/services/PlasmidFinder/) and PLACNETw [30] (https://castillo.dicom.unican.es/upload/). Default parameters were used for all software.

For comprehensive genomic analysis, we used BacWGSTdb (http://bacdb.org/BacWGSTdb), which allowed us to find the closest isolates that are currently deposited in the GenBank database [27]. The whole-genome sequences of 37 closely related to our *A. pittii* strains were downloaded from the GenBank database. The genomic comparison was conducted using a reference genome-based single nucleotide polymorphism (SNP) strategy with CSI Phylogeny [31]. The result was constructed phylogenetic trees using MEGA-X, via the neighbor-joining method with 500 bootstrap replicates by applying the Tamura three-parameter model [21]. The phylogenetic tree was visualized using the Interactive Tree of Life (iTOL) (http://itol.embl.de) [32].

### 2.8. Statistical Analysis

The clinical characteristics of XDRAP were analyzed by comparing with XDR *A. baumannii* (XDRAB), also collected during the current study. Of the total 832 CRACB cases, 6 were XDRAP, while 18 were XDRAB. Clinical data of these cases were analyzed by logistic regression using Stata version 12.0 software (StataCorp, College Station, TX, USA). Data were considered significant at *p* < 0.05.

### 2.9. Nucleotide Sequence Accession Numbers

The assembled genomic sequences were deposited in the NCBI Genbank Database under the Bioproject accession number of PRJNA602201.

## 3. Results

### 3.1. Identification, Susceptibility, and Genotyping

The studied criteria included only carbapenem-resistant *Acinetobacter calcoaceticus-baumannii* complex (CRACB). Of the total 832 carbapenem-resistant CRACB isolates used in this study, 826 were identified as *A. baumannii* (99.3%), and 6 (0.7%) were identified as *A. pittii*. Among the 826 *A. baumannii*, 18 isolates were XDR (2.2%). All the *A. pittii* isolates in this study were resistant to carbapenem and showed presence of *bla*_NDM-1_, *bla*_IMP-1_, and *bla*_IMP-14_ genes, as well as oxacillinase genes like *bla*_OXA-10_, *bla*_OXA-58_, and *bla*_OXA-23_. Table 1 shows the clinical data of these six patients, of which five were male (83%) and one female (17%), with an age range of 19–73 years. Three cases were classified as hospital-acquired infections, whereas the rest were classified as colonization. Five of the six patients survived, while no data were available for one case.

The results of antimicrobial susceptibility tests are shown in Table 2. All carbapenem-resistant *Acinetobacter pittii* (CRAP) isolates were resistant to ceftazidime, cefepime, cefotaxime, ceftriaxone, doripenem, imipenem, meropenem piperacillin, and trimethoprim–sulfamethoxazole. All the isolates were intermediately resistant to colistin. Three isolates were found to be susceptible to gentamicin and amikacin, while four isolates were susceptible to ciprofloxacin and tetracycline. Five isolates were identified as extensively drug-resistant (XDR) which is defined according to the guideline described elsewhere [33].

We confirmed the presence of carbapenemase genes by multiplex PCR and sequencing. All isolates carried both oxacillinase and metallo-β-lactamase genes (Table 1). The *bla*_NDM-1_ was detected among the MBLs genes (3/6; 50%), while *bla*_OXA-58_ was predominant among oxacillinase genes (3/6; 50%), followed by *bla*_OXA-10_ (2/6; 33.3%). Plasmid typing based on PCR revealed all CRAP isolates carried at least 1 plasmid group (GR) and a maximum of 4 GRs out of 19 groups of rep genes (GR12, GR8 and GR3) were mostly found in 5 out of 6 isolates (83.3%).

**Figure 1:**
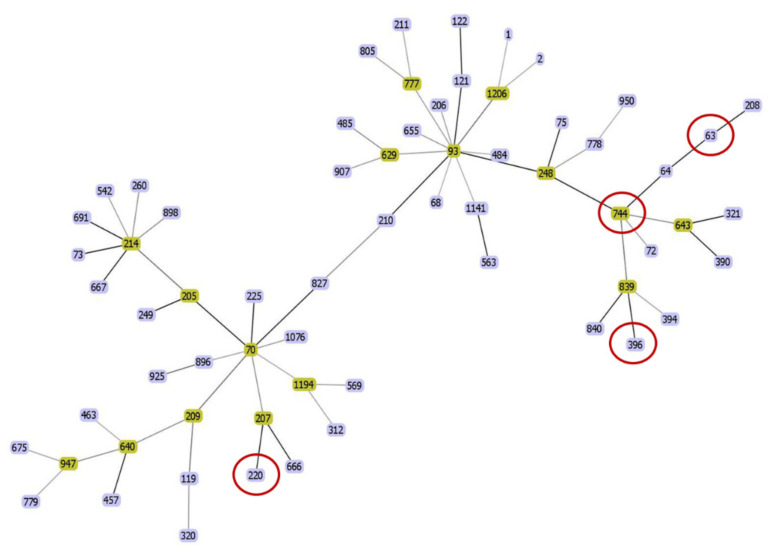
Minimum spanning tree of sequence types (ST) of 65 *A. pittii*, constructed with goeBURST. The seven CRAP isolates belonging to four STs are denoted as red circles.

MLST analysis revealed that six CRAP isolates belonged to four STs: two (A864 and A984) were assigned to ST220, two (A56 and A273) were ST744, and one each belonged to ST396 (A1) and ST63 (A436), respectively, according to the Pasteur scheme (Table 1). The goeBURST displayed a clonal complex of CRAP, as shown in Figure 1. ST396 and ST744 were closely related to ST839, whereas ST220 was related to ST207. ST63 was related to ST64 and ST208. A phylogenetic tree was constructed using the concatenated sequence of four STs as shown in Figure S1. It demonstrated that ST63 was closely related to ST208, while ST744 was closely related to ST122 and ST121. ST396 was closely related to ST839 and ST840. ST220 was related to ST207, ST666, ST227, and ST1206

### 3.2. Genomic Characterization of Extensively-Drug Resistant A. pittii

**Figure 2:**
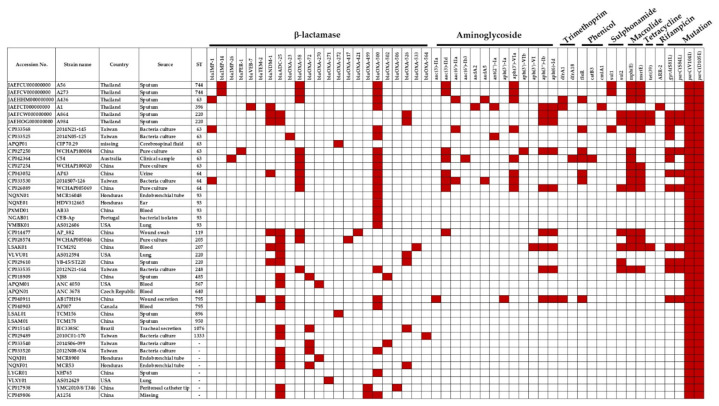
Genomic characterization of antibiotic-resistant genes in carbapenem-resistant *A. pittii.* The present of antimicrobial resistance genes is represented in red box.

The draft genome sequence of six CRAP isolates is shown in Table S3 in the Supplementary Materials. As shown in Figure 2, three isolates of CRAP (A56, A273, and A436 strain) carried *bla*_IMP_ and the following: β-lactamase resistance genes (*bla*_OXA58_ and *bla*_OXA500_), aminoglycoside resistance genes (*aac(*3*)-IId*, *aac(*6*′)-IIa*, *aadA*2, and *aph(*3*′)VIa*), sulphonamide resistance gene (*sul*1), trimethoprim resistance gene (*dfrA*1), phenicol resistance gene (*floR*), and macrolide resistance genes (*mph*(E) and *msr*(E)). The remaining 3 CRAP (A1, A864 and A984 strain) isolates harbored *bla*_NDM-1_ and the following: β-lactamase resistance genes (*bla*_VEB-7_, *bla*_ADC-25_, *bla*_OXA500_ and *bla*_OXA526_), aminoglycoside resistance genes (*aadA*2, *ant(*2*″)-Ia*, *aph(*3*″)Ib*, and *aph(*6*)Id*), sulphonamide resistance gene (*sul*2), trimethoprim resistance gene (*dfrA*1), tetracycline (tet39), phenicol resistance gene (*cmlA*1), rifampicin (*ARR-*2), and macrolide resistance genes (*mph*(E) and *msr*(E)). All CRAP isolates contained a mutation of *parC* gene (Figure 2).

Analysis of the acquired antibiotic-resistant genes showed that isolate A436 harbored 10 antimicrobial resistance genes including *dfrA1*, *aac*(6*′)-lla*, *aadA*5, *aph(*3*′)-VIa*, *bla*_IMP-1_, *bla*_OXA58_, and *bla*_OXA-500_, as well as *floR*, *mph(E)*, and *msr(E)*. Similarly, isolate A1 also revealed 10 resistant genes but differed from isolate A436 in some genes: *bla*_VEB-7_, *bla*_NDM-1_, *bla*_OXA-500_, *aadA*2, *ant(*2*″)-Ia*, *aph(*3*′)-Ib*, *aph(*6*)Id*, *dfrA*1, *cmlA*1, and *ARR-*2 (Figure 2). Isolates A984 and A864 carried 9 antimicrobial resistance genes, including *aph(*3*″)-lb*, *aph(*6*)-ld)*, *bla*_NDM-1_, *bla*_OXA526_, *bla*_ADC-25_, *sul2*, *mph(E)*, *msr(E)*, and *tet(39)*, whereas isolates A56 and A273 contained 8 resistant genes, including *bla*_IMP-14_, *bla*_OXA-58_, *bla*_OXA-500_, *aac(3)-IId*, *aph(*3*′)-VIa*, *sul1*, *mph(E)*, and *msr(E)* (Figure 2). The *bla*_NDM-1,_ *bla*_ADC-25_, and *bla*_OXA-526_ were present in the ST220 isolates (A984, A864 YB-45, AS012594). Similarly, two ST63 isolates, A436 and WCHAP100020, carried *bla*_OXA-500_ and *bla*_OXA-58_, respectively. However, *bla*_IMP-1_ was also found in A436. In addition, resistance–nodulation–cell division (RND) antibiotic efflux pump genes (*adeF*, *adeL*), major facilitator superfamily (MFS) antibiotic efflux pump genes (*amvA*, *abaQ*, *floR*), and small multidrug resistance (SMR) antibiotic efflux pump genes (*abeS*) were also present in these six isolates. These pumps are responsible for fluoroquinolone, macrolide, tetracycline, and phenicol efflux.

Plasmid analysis of genomic sequences of the six isolates using PlasmidFinder revealed no Inc group replicons. However, PLACNETw showed 4 MOB plasmid types; MOBQ in 4 isolates (A1, A436, A864, A984), MOBV in 2 isolates (A56, A273), MOBP in 2 isolates (A864, A984), and MOBF in the A436 isolate. Three isolates (A436, A864, A984) contained 2 MOB plasmid types.

**Figure 3:**
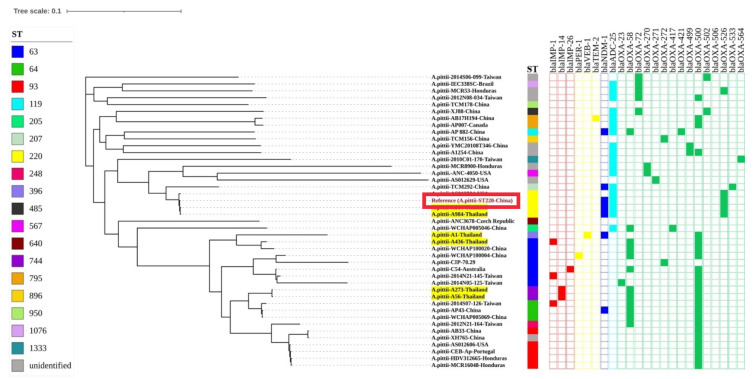
Whole-genome phylogeny analysis of *A. pittii* generated by CSI Phylogeny and visualized with interactive tree of life tool. The whole genome sequence of *A. pittii* in our studies is shown in yellow highlight and *A. pittii*-ST220-China as a reference genome is denote in red square box. Sequence type (STs) and β-lactamase genes are shown in each isolate. The filled symbols reveal the presentation of the genes, whereas unfilled symbols reveal their absence.

As shown in Figure 3, the whole-genome SNP using CSI Phylogeny revealed that isolates A984 and A864 were closely related to the reference *A. pittii* YB-45 (ST220) isolate from China, recovered from sputum, while isolate A436, which was related to *A. pittii* strain WCHAP100020, was isolated from China. By contrast, A273 and A56 isolates were clustered together; these isolates were related to strains 2014S07-126, AP43, WCHAP005069, which were isolated from Taiwan and China. The isolate A1 was clustered together with our isolate A436 and WCHAP100020; however, it is located at a different branch.

### 3.3. Clinical Analysis

We compared 18 XDRAB cases and 6 XDRAP cases for demographic association by logistic regression. For a total of 24 cases, mean age for analysis was 59.52 (SD = 3.65; min = 19 years old; max = 82 years old), with 19 males (82.6%) and 4 females (17.4%). Univariate analysis did not show any correlation. Multivariate analysis also showed no correlation between XDRAP and XDRAB concerning the elderly (OR = 1.3; 95% CI = 0.09−12.96; *p* = 0.586) and male (OR = 1.00; 95% CI = 0.16−16.30; *p*-value = 0.71) cases. In addition, antibiotic usage, length of hospitalization, and predisposing conditions did not show any correlation.

## 4. Discussion

Over the last decade, the presence of carbapenemase-producing *A. pittii* has become dominant in several countries, and it is being increasingly considered a nosocomial pathogen [34,35]. A previous study in Thailand revealed that 6.4% (22/346) were *A. pittii*, of which 22.7% (5/22) were carbapenem-resistant [8]. Our study revealed 0.7% of *A. pittii* in a hospital in rural Thailand (lower than that reported previously), but all of them were carbapenem-resistant. All the patients survived. XDRAP showed a correlation with male and elderly patients; however, the small number of XDRAPs observed in this study limited their analysis. A retrospective study conducted at a teaching hospital in Taiwan revealed that the 14-day and 28-day mortality rates of *A. pittii* bacteremia were 14% and 17%, respectively [36]. A study in Thailand demonstrated that patients infected with carbapenem-susceptible *A. nosocomialis* and *A. pittii* had lower 30-day mortality than those infected with carbapenem-susceptible *A. baumannii* and carbapenem-resistant *A. baumannii* [37]. Moreover, a recent study demonstrated that *A. seifertii* and *A. pittii* presented higher pathogenicity in in vitro and in vivo models than *A. baumannii* and *A. nosocomialis* [38].

The common carbapenemase genes present in CRAP are *bla*_OXA-23_ and *bla*_OXA-58_ [39,40,41,42]. MBL genes such as *bla*_IMP-1_, *bla*_IMP-4_, *bla*_IMP-19_, and *bla*_NDM-1_ were also detected in CRAP [43,44,45,46,47,48,49]. Coexistence of oxacillinase and MBLs genes in *A. pittii* has been reported in Australia; *bla*IMP-4 and *bla*_OXA-96_ [50] in Japan; *bla*_IMP-1_, and *bla*_OXA-58_ [51] in Malaysia; *bla*_NDM-1_ and *bla*_OXA-58_ [10] and Thailand; *bla*_IMP-14a_ and *bla*_OXA-58_ [8]. Our study found that all the CRAP isolates harbored either *bla*_OXA-series_, *bla*_NDM-1_, *bla*_IMP-65-like_, or *bla*_IMP-1_. This is the first report of the presence of *bla*_NDM-1_ in CRAP found in Thailand. This suggests that dissemination of *bla*_NDM-1_ may occur among the Enterobacteriaceae *A. pittii*, and *A. baumannii*. In addition, *A. pittii* may play a role in the dissemination of *bla*_NDM-1_ to Enterobacteriaceae [52].

In the present study, four STs (ST63, ST220, ST396, and ST744) were assigned to CRAP, of which ST220 was the most predominant. This ST was reported in Japan and China, and carried *bla*_NDM-1_, like our isolate [4,53]. ST744 was the second most predominant ST in this study; it was found in Germany from the MLST database (https://pubmlst.org/bigsdb?page=profileInfo&db=pubmlst_abaumannii_pasteur_seqdef&scheme_id=2&profile_id=744). ST63 was reported in Japan, Korea, and China [11,54,55]. ST396 was also reported in Korea [11]. Interestingly, ST220 seems to the most susceptible to aminoglycoside agents. Our study showed that 66.6% (2 isolates) of ST220 were susceptible to netilmicin, gentamicin, and amikacin. Two ST220 isolates reported elsewhere revealed that *A. pittii* SU1805 (ST220), isolated from a hospital sink in Japan, was susceptible to gentamicin and amikacin, whereas *A. pittii* YB-45 from China was susceptible to gentamicin and tobramycin [4,53].

Whole-genome sequences of *A. pittii* have been reported in ST119, ST207 (strain TCM292), ST220 (strain YB-45), ST865 (strain TCM156), and several strains deposited in GenBank [44,53,56,57]. Whole-genome SNP phylogeny revealed that our XDRAP isolates showed that the A436 (ST63) isolate was closely related to the strain WCHAP100020 from China. The XDRAP isolates A984 and A864 (ST220) were clustered with strain YB-45/ST220 from China and strain ASO12594 from the United States of America. A56 and A273 isolates were clustered together and are closely related to strains 2014S07-126, AP43, and WCHAP005069, isolated from Taiwan and China. Isolate A1 (ST396) was clustered together with isolates A436 and WCHAP100020. However, all of them have common ancestors for each cluster. Whole-genome sequencing is a powerful tool for source tracking, surveillance monitoring, and dynamic populations.

*Acinetobacter baumannii* is of concern to the World Health Organization because it resists most commercially available antibiotics and causes hospital-acquired infections. Increasing numbers of multidrug-resistant *A. pittii* and XDRAP worldwide require strengthening of official surveillance and close monitoring in order to prevent outbreaks and contain the spread in parallel with *A. baumannii.*

## Figures and Tables

**Table 1:** Clinical features of 6 *Acinetobacter pittii* isolates carrying carbapenemase gene.

Isolate No.	Specimen	Age	Sex	Status	Disease	Underlying Disease	Outcome	ST	Genes		Plasmids
									OXA	MBL	
A1	Sputum	71	M	Colonization	Ischemic heart disease with Atrial fabulation with Staphylococcus	Ischemic heart disease	Survive	396	23, 51	NDM-1	GR12, GR8
A56	Sputum	75	M	Colonization	Fever of unknown origin	Unknown	Unknown	744	58	IMP-14	GR3, GR12, GR8, GR16
A273	Sputum	66	M	Colonization	Hypotension with Pneumonia with CKD-5 * with DM *	CKD-5 * with DM *	Survive	744	58	IMP-14	GR3
A436	Sputum	34	F	Infection	Heart failure with Respiratory failure with Atrial fabulation with Hypertension with Bacterial pneumonia	Hypertension	Survive	63	58	IMP-1	GR3, GR12, GR8, GR6
A864	Sputum	46	M	Infection	Gastroenteritis with DM *	DM *	Survive	220	10	NDM-1	GR3, GR12, GR8
A984	Ascitic Fluid	73	M	Infection	CKD-5 * with Gout with Hypertension Migraine with Liver cell carcinoma with Ascitic	CKD-5 * with Gout with Hypertension with CA Liver	Survive	220	10	NDM-1	GR3, GR12, GR8

* CKD; chronic kidney disease stage 5, DM; Diabetes mellitus.

**Table 2:** Antimicrobial susceptibility profiles of carbapenem resistant *A. pittii*.

Isolate No.	MIC (µg/L)													Disk Diffusion Assay (mm)			
	CAZ	FEP	CTX	CRO	DOR	IPM	MEM	CL	GM	AMK	NET	CIP	SXT	TE	TZP	PIP	
A1	>32	>32	>32	>32	>4	>8	>8	≤1	≤2	≤8	≤8	0.12	>4	20	12	11	
	(R)	(R)	(R)	(R)	(R)	(R)	(R)	(I)	(S)	(S)	(S)	(S)	(R)	(S)	(R)	(R)	MDR
A56	32	32	>32	>32	>4	>8	>8	≤1	>8	>32	≤8	≤0.06	>4	20	19	18	
	(R)	(R)	(R)	(R)	(R)	(R)	(R)	(I)	(R)	(R)	(S)	(S)	(R)	(S)	(R)	(R)	XDR
A273	16	32	>32	>32	>4	>8	>8	≤1	>8	>32	>16	1	>4	20	17	16	
	(I)	(R)	(R)	(R)	(R)	(R)	(R)	(I)	(R)	(R)	(I)	(S)	(R)	(S)	(R)	(R)	XDR
A436	>32	32	>32	>32	>4	>8	>8	≤1	8	>32	≤8	0.12	>4	23	18	16	
	(R)	(R)	(R)	(R)	(R)	(R)	(R)	(I)	(I)	(R)	(S)	(S)	(R)	(S)	(I)	(R)	XDR
A864	32	>32	32	>32	>4	>8	>8	≤1	≤8	≤8	≤8	>2	>4	9	12	12	
	(R)	(R)	(R)	(R)	(R)	(R)	(R)	(I)	(S)	(S)	(S)	(R)	(R)	(R)	(R)	(R)	XDR
A984	32	>32	32	>32	>4	>8	>8	≤1	4	≤8	≤8	>2	>4	7	12	12	
	(R)	(R)	(R)	(R)	(R)	(R)	(R)	(I)	(S)	(S)	(S)	(R)	(R)	(R)	(R)	(R)	XDR

CAZ: Ceftazidime; FEP: Cefepime; CTX: Cefotaxime; CRO: Ceftriaxone; DOR: Doripenem; IPM: Imipenem; MEM: Meropenem; CL: Colistin; GM: Gentamicin; AMK: Amikacin; NET: Netimicin; CIP: Ciprofloxacin; SXT: Trimethoprim–sulfamethoxazole; TE: Tetracyclin; TZP: Piperacillin–tazobactam; PIP: Piperacillin.

## Data Availability

The results of this whole-genome shotgun project were deposited in DDBJ/ENA/GenBank under the BioProject no. PRJNA602201, BioSample no. SAMN17073293 for A1, SAMN17073294 for A56, SAMN17073295 for A273, SAMN17083170 for A436, SAMN17073296 for A864 and SAMN17083171 for A984, and accession no. JAEFCT000000000 for A1, JAEFCU000000000 for A56, JAEFCV000000000 for A273, JAEHHM000000000 for A436, JAEFCW000000000 for A864 and JAEHOG000000000 for A984.

## Supplementary Materials

The following are available online at https://www.mdpi.com/2076-2607/9/2/242/s1, Figure S1: Phylogenetic analysis of concatenated sequences of 7 MLST genes of *A. pittii*., Table S1: Primers sequences for detection Acinetobacter species and carbapenem resistant genes, Table S2: Primers sequences for detection of plasmid typing, Table S3: Genome information of carbapenem resistance *Acinetobacter pittii*.
