# Supplementary material for: Genomic Characterization of Clinical Extensively Drug-Resistant Acinetobacter pittii Isolates
Source: Microorganisms. 2021 Jan 25;9(2):242. doi: 10.3390/microorganisms9020242 (PMC7912037; doi:10.3390/microorganisms9020242)
Supplement: Supplementary file 1 [file microorganisms-09-00242-s001.pdf]

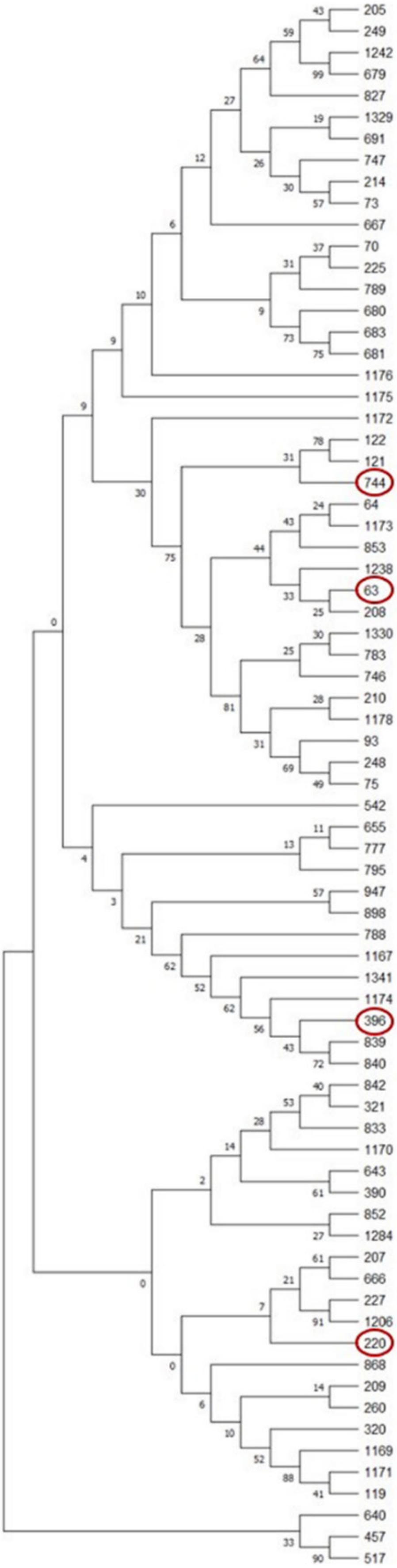
205
249
1242
679
827
1329
691
747
214
73
667
70
225
789
680
683
681
1176
1175
1172
122
121
744
64
1173
853
1238
63
208
1330
783
746
210
1178
93
248
75
542
655
777
795
947
898
788
1167
1341
1174
396
839
840
842
321
833
1170
643
390
852
1284
207
666
227
1206
220
868
209
260
320
1169
1171
119
640
457
517

Supplementary Figure S1. Phylogenetic analysis of concatenated sequences of 7 MLST genes of *A. pittii*. A phylogenetic tree was constructed from the concatenated sequence of the MLST allelic loci using the neighbor-joining method with MEGA-X software. The seven CRAP isolates belonging to four STs are denoted as. The red circles shown STs were found in our studied.

Supplementary TableS1 Primers sequences for detection Acinetobacter species and carbapenem resistant genes

Genes	Primer sequence (5′-3′)	References
D14	GACAACAGTTATAAGGTTTCAGGTG	13
D19	CCGCTATCTGTATCCGCAGTA	
D16	GATAACAGCTATAAAGTTTCAGGTGGT	
D8	CAAAAACGTACAGTTGTACCACTGC	
Sp2F	GTTCCTGATCCGAAATTCTCG	
Sp4F	CACGCCGTAAGAGTGCATTA	
Sp4R	AACGGAGCTTGTCAGGGTTA	
OXA-51-like	F-TAATGCTTTGATCGGCCTTG	16
	R-TGGATTGCACTTCATCTTGG	
OXA-23-like	F-GATCGGATTGGAGAACCAGA	
	R-ATTTCTGACCGCATTTCCAT	
OXA-24-like	F-GGTTAGTTGGCCCCCTTAAA	
	R-AGTTGAGCGAAAAGGGGATT	
OXA-58-like	F-AAGTATTGGGGCTTGTGCTG	
	R-CCCCTCTGCGCTCTACATAC	
OXA-10-like	F-TTAGGCCTCGCCGAAGCG	17
	R-CTTTGTTTTAGCCACCAATGATG	
IMP	F-GGAATAGAGTGGCTTAAYTCTC	18
	R- GGTTTAAYAAAACAACCACC	
OXA48-like	F- GCGTGGTTAAGGATGAACAC	
	R- CATCAAGTTCAACCCAACCG	
NDM	F- GGTTTGGCGATCTGGTTTTC	
	R- CGGAATGGCTCATCACGATC	
KPC	F- CGTCTAGTTCTGCTGTCTTG	
	R- CTTGTCATCCTTGTTAGGCG	
MCR1	F- GGGTGTGCTACCAAGTTTGC	
	R- CATTGGCGTGATGCCAGTTT	

Supplementary TableS2 Primers sequences for detection of plasmid typing

Name	Primer sequence (5′-3′)	References
gr1FW	F-CATAGAAATACAGCCTATAAAG	19
gr1RV	-RTTCTTCTAGCTCTACCAAAAT	
Gr2FW	F-AGTAGAACAACGTTTAATTTTATTGGC	
Gr2RV	R-CCACTTTTTTTAGGTATGGGTATAG	
Gr3FW	F-TAATTAATGCCAGTTATAACCTTG	
Gr3RV	R-GTATCGAGTACACCTATTTTTTGT	
Gr5FW	F-AGAATGGGGAACTTTAAAGA	
Gr5RV	R-GACGCTGGGCATCTGTTAAC	
Gr18FW	F-TCGGGTTATCACAATAACAA	
Gr18RV	R-TAGAACATTGGCAATCCATA	
Gr7FW	F-GAACAGTTTAGTTGTGAAAG	
Gr7RV	R-TCTCTAAATTTTTCAGGCTC	
Gr9FW	F-GCAAGTTATACATTAAGCCT	
Gr9RV	R-AAAAATAAACGCTCTGATGC	
Gr4FW	F-GTCCATGCTGAGAGCTATGT	
Gr4RV	R-TACGTCCCTTTTTATGTTGC	
Gr11FW	F-GGCTATTCAAAACAAAGTTAC	
Gr11RV	R-GTTTCCTCTCTTACACTTTT	
Gr12FW	F-TCATTGGTATTCGTTTTTCAAAACC	
Gr12RV	R-ATTTCACGCTTACCTATTTGTC	
Gr10FW	F-TTTCACTAGCTACCAACTAA	
Gr10RV	R-ACACGTTGGTTTGGAGTC	
Gr13FW	F-CAAGATCGTGAAATTACAGA	
Gr13RV	R-CTGTTTATAATTTGGGTCGT	
Gr8FW	F-AATTAATCGTAAAGGATAATGC	
Gr8RV	R-GACATAGCGATCAAATAAGC	

Gr14FW	F-TTAAATGGGTGCGGTAATTT	
Gr14RV	R-GCTTACCTTTCAAAACTTTG	
Gr15FW	F-GGAAATAAAAATGATGAGTCC	
Gr15RV	R-ATAAGTTGTTTTTGTTGTATTCG-	
Gr16FW	F-CTCGAGTTCAGGCTATTTTT	
Gr16RV	R-GCCATTTCGAAGATCTAAAC	
Gr17FW	F-AATAACACTTATAATCCTTGTA	
Gr17RV	R-GCAAATGTGACCTCTAATATA	
Gr6FW	F-AGCAAGTACGTGGGACTAAT	
Gr6RV	R-AAGCAATGAAACAGGCTAAT	
Gr19FW	F-ACGAGATACAAACATGCTCA	
Gr19RV	R-AGCTAGACATTTCAGGCATT	

Supplementary TableS3. Genome information of carbapenem resistance *Acinetobacter pittii*

Sample	Total length (bp)	N50 length (bp)	Max length (bp)	Min length (bp)	GC content (%)	Average length (bp)
A1	4380367	216817	503904	281	38.67	62576.67
A56	4034194	88018	402818	281	38.78	33618.28
A273	3970201	91064	215838	281	38.79	33933.34
A436	4,204,171	72,982	272,977	365	38.6	545981
A864	4034895	79364	243857	281	38.81	58526.61
A984	4,127,158	75,037	231,589	281	38.79	535843
